# Similarity-Dissimilarity Competition in Disjunctive Classification Tasks

**DOI:** 10.3389/fpsyg.2013.00026

**Published:** 2013-02-08

**Authors:** Fabien Mathy, Harry H. Haladjian, Eric Laurent, Robert L. Goldstone

**Affiliations:** ^1^Department of Psychology, Université de Franche-ComtéBesançon, France; ^2^School of Social Sciences and Psychology, University of Western SydneySydney, NSW, Australia; ^3^Department of Psychological and Brain Sciences, Indiana UniversityBloomington, IN, USA

**Keywords:** learning, categorization, disjunctive rules, dimension saliency, similarity effects, rule-based, exemplar-based, hybrid

## Abstract

Typical disjunctive artificial classification tasks require participants to sort stimuli according to rules such as “*x* likes cars only when black and coupe OR white and SUV.” For categories like this, increasing the salience of the diagnostic dimensions has two simultaneous effects: increasing the distance between members of the same category and increasing the distance between members of opposite categories. Potentially, these two effects respectively hinder *and* facilitate classification learning, leading to competing predictions for learning. Increasing saliency may lead to members of the same category to be considered *less* *similar*, while the members of separate categories might be considered *more* *dissimilar*. This implies a similarity-dissimilarity competition between two basic classification processes. When focusing on sub-category similarity, one would expect more difficult classification when members of the same category become less similar (disregarding the increase of between-category dissimilarity); however, the between-category dissimilarity increase predicts a less difficult classification. Our categorization study suggests that participants rely more on using dissimilarities between opposite categories than finding similarities between sub-categories. We connect our results to rule- and exemplar-based classification models. The pattern of influences of within- and between-category similarities are challenging for simple single-process categorization systems based on rules or exemplars. Instead, our results suggest that either these processes should be integrated in a hybrid model, or that category learning operates by forming clusters within each category.

## Introduction

The idea that perception influences categorization has considerable intuitive appeal (Goldstone and Barsalou, 1998). It has long been shown that categorization performance can be adequately described as a function of the cohesion between stimuli (or statistical density), which can be measured by a simple within- and between-category distance ratio (Homa et al., 1979; Sloutsky, 2010). Little is known, however, about the effect of category cohesion in disjunctive classification tasks for which objects do not perceptually or conceptually resemble one another. A disjunction is a logical term that expresses a close connection to the *or* word in natural languages. For instance, one can express the idea of preferring “a large house in the suburbs *or* a small apartment downtown”, as opposed to “large houses downtown (that you could not afford) *or* small apartments in the suburbs (that would be of no interest because you can afford a better place)^1^.” Ultimately, the positive examples of a disjunctive category share no characteristics that are common to all its members. Studies in the 1950s showed that learning strategies are most often based on positive information, that is, they avoid using cues in the stimuli that tell them “what the object is not” (to rephrase Bruner et al., 1956, chap. 6, p. 181). The absence of a shared characteristic in disjunctive classification tasks impedes learning strategies and also undermines the role of similarity in subserving disjunctive categorizations (Goldstone, 1994b).

Generally, there are two ways of organizing the category learning process. The first is to find differences between categories, a process that has recently been emphasized by researchers who argue that a category learner needs to find features that discriminate between categories (Kornell and Bjork, 2008). For example, people traditionally try to define male and female by observing differences in behavior and personality between genders. An alternate method is to find similarities between sub-categories and develop a positive, stand-alone representation of each category. For example, in educational settings, students acquire formal concepts from instruction that defines a set of properties applying to a class of objects, such as “a square has four equal sides and at least one 90°.” Occasionally, case-based reasoning is sufficient to provide adequate knowledge (e.g., you do not need to hear many gun shots to grasp the relationship between fire and noise). Reasoning by analogy is another example in which similarity applies predominantly.

If this broad organization is correct, then there are two situations that could make learning a category difficult. Two separate categories might both have items that are very similar. For example, this is the problem facing the friend of two identical twins, Karen and Sharon. There is the Karen category of all of the variations in appearance that Karen can assume (e.g., on different days with different lighting). Here, the basic category learning difficulty is that this set of appearance possibilities is very similar to the set of appearances that instances of the Sharon category can assume. Another situation that can make categorization difficult is when it is hard to find anything that the members of a category share because within-category similarity is so low. This is one of the reasons why the mammal category is harder to learn than the dog category – things that are at first glance quite dissimilar, like whales, bats, and rabbits, all share some subtle commonalities that place them in the mammal category.

Most research in the spirit of the present study has focused on one-dimensional rules, which would imply, for instance, considering only the price of a property regardless of its location (e.g., Goldstone and Steyvers, 2001; Goldstone et al., 2001). However, disjunctive categories present the peculiar effect of simultaneously increasing the psychological distance between members of the same category and increasing the distance between members of separate categories when the salience of the categorization-relevant dimensions increase. Such an effect is related to the more general phenomenon that category intuitiveness, which is influenced by the tightness of clusters and the separation between clusters (Pothos et al., 2011), influences categorization processes. In other words, increasing the salience of a relevant feature dimension will increase the perceived dissimilarity between two members of the same category as well as members of two different categories. Potentially, these two effects can respectively hinder and facilitate classification learning, thus implying a similarity-dissimilarity competition between two classification processes, that is, finding similarities between sub-categories and highlighting differences between categories (see Stewart and Morin, 2007). More precisely, the similarity-dissimilarity competition terminology that we employ specifically refers to a Less-similarity-within-categories versus a More-dissimilarity-between-categories competition process. For example, imagine someone has a preference for either perfectly white cats or perfectly black dogs, rather than white dogs or black cats. The large differences between the categories (white and black are highly dissimilar, and cats and dogs are quite dissimilar) might help one learn to distinguish the categories (favorite pets versus non-favorite pets), relative to learning categories that are closer (in which all of the objects are less distinguishable). Conversely, these exact same differences interfere with determining commonalities between the white cats and the black dogs that belong to the same category of “favorite pets.” Given that individuals generally look for underlying common features or causes (e.g., Rehder and Kim, 2009) in category learning (such as cleaning the white fur is a burden, or cats are more independent), the within-category dissimilarities imposed by the disjunctive categories hinder their acquisition. To limit the terminology throughout the paper, only three entities will be opposed in the present study: distance between categories, distance between sub-categories, and distance within sub-categories. The reason is that within categories, individuals can either seek similarities between objects of different sub-categories (also called clusters), or between objects within sub-categories. Our terminology simply avoids, for instance, formulations such as “ within-category between cluster” or “ between-category between cluster.”

One purpose of our study was to further test the exemplar model (Nosofsky, 1986), in which categorization accuracy is determined by the ratio of evidence in favor of one category to the sum of the evidence in favor of all possible categorizations. In exemplar models, both within-category and between-category similarities affect categorization accuracy. Thus, a manipulation that draws all stimuli closer to one another might be expected to impair categorization according to some exemplar models. This prediction will be formalized in a later section. A second prediction was that seeking similarities between members of a category is more typical of a rule-based process (considering that a rule is usually based on positive information; e.g., Feldman, 2000), so a manipulation that draws all stimuli closer to one another might be expected to improve rule-based categorization.

Foreshadowing our main result, this study shows that the similarity-dissimilarity competition results in participants relying more on the dissimilarities between opposite categories rather than finding similarities between sub-categories. This learning strategy makes disjunctive classification learning less difficult as the distance between sub-categories increases. These results give us an opportunity to discuss rule-versus exemplar-based accounts and to propose an interpretation based on the more flexible SUSTAIN model.

**Figure 1:**
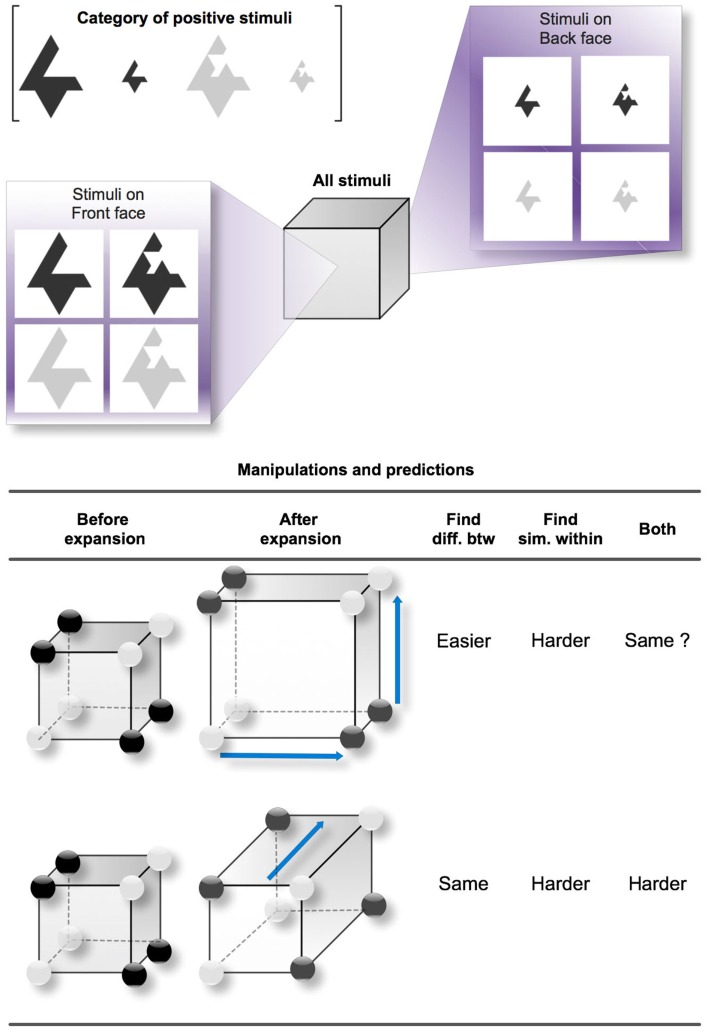
**Studied variations of distances between the positive examples of a Type II concept**. The reference cube exemplifies a Type II concept with the Size dimension irrelevant. The three dimensions of the cube respectively represent Size (front face versus back face), Shape (left face versus right face), and Color (top face versus bottom face). One edge in the cube represents one difference in features. For instance, between the top left vertex on the front face (a large and simple dark object) and the bottom right vertex on the back face (a small and complex light object), there is a (city-block) distance of three edges, expressing the fact that the two objects differ in three dimensions. The dark circles placed on certain vertices denote the positive examples of the Type II concept. Here, the correct rule is (*simple* and *dark*) or (*complex* and *light*). The two study cases (one study case per line) correspond to two variations of the distances between the positive examples that we studied. In the first case, the two relevant dimensions become more salient, so the perceived distance between the clusters becomes greater. In the second case, the irrelevant dimension becomes more salient, making the distances between the objects within clusters greater. The predictions are indicated for two different processes: finding differences between categories and finding similarities between clusters (as well as both combined).

Figure 1 shows the possible variations of feature distances that we manipulated in our study and the corresponding predictions for two distinct category learning processes: finding differences between categories and finding similarities between sub-categories, as well as a combination of both. In Figure 1, we chose a three-dimensional (3D) “Size-irrelevant/Color-and-Shape-relevant” Type II concept to serve as an example (the positive category is formed by the two simple dark objects *or* the two complex light objects, regardless of their size). Type II classification tasks were originally studied by Shepard et al. (1961) along with five other types that we do not describe here. In Type II classifications, two out of the three dimensions are diagnostic to solving the problem, while information about the third dimension is irrelevant. The structure of Type II is thus derived from the simpler 2D exclusive OR (i.e., XOR), although the presence of the irrelevant dimension can complicate learning. We chose to experiment on Type II classifications because they are the simplest disjunctive problems over three dimensions in the Shepard et al.’s classification (see also Nosofsky et al., 1994a). Another advantage in Type II concepts is the parity between positive and negative examples (four of each kind), which makes the disjunction symmetrical for positive and negative examples, so it does not matter whether participants focus on positive or negative examples.

In our example, the Type II concept applies to three Boolean-valued dimensions of shape, size, and color, which produce eight possible stimuli when combined (from the large dark complex shape to the small light simple shape). Each of the eight stimuli can be reported on one vertex of a cube, following a city-block distance between the stimuli. For instance, two stimuli differing only in one feature are separated by one edge, two stimuli differing in two features are separated by two edges, etc. In Figure 1, the cube contrasts the large and small stimuli using respectively the front face and the back face, the dark and the light stimuli using the top and bottom faces, and the simple and complex shapes using the left and right faces. Generally, a set of dark circles is apposed to the cube in order to indicate which of the stimuli belong to the positive category; the other stimuli, represented by white circles, belong to the negative category. For the Type II concept that we arbitrarily chose in this example, the positive category gathers the simple dark shapes and the complex light shapes. The simplest rule for distinguishing the categories is thus: “The object is positive IF (simple *and* dark) *or* (complex *and* light).” Such a rule has a disjunctive normal form because it is a disjunction of conjunctive clauses. This Type II concept therefore comprises four clusters or sub-categories: simple *and* dark objects, complex *and* light objects, complex *and* dark objects, and the simple *and* light objects.

The bottom table in Figure 1 reports two study cases (one per line) that lead to different predictions. In the first case, the distance between the values of the two relevant dimensions increases and is expressed by the expansion of the cube for both relevant dimensions. This makes the positive examples more separable from the negative ones, but at the same time increases the dissimilarity between the two positive sub-categories of the positive category. In the second case, the values for the irrelevant dimension become more distant, so the incentive for grouping the positive examples within sub-categories is lower.

We now describe a simplified version of the generalized context model (GCM) called the raw similarity model (RSM) because both make the same predictions for our study cases. RSM predicts that the difficulty of category learning will be based on finding differences between categories. Effectively, RSM computes the probability of putting each example in each category, assuming that the classification of an example is determined by its similarity to the stored category exemplars (Medin and Schaffer, 1978; Nosofsky, 1984). Every seen exemplar of the categories is compared to an instance that must be categorized. These exemplars are the psychological representatives of the corresponding concrete stimuli. A simple distance function was used with a city-block metric (counting the number of different features between two stimuli), *n* the number of dimensions composing the stimuli (here, *n* = 3), and *x_ia_* the value of stimulus *i* on dimension *a*:

(1) $$d_{ij}=\overset{n}{\underset{a=1}{\sum }}\left|x_{ia}-x_{ja}\right|$$

The following exponential decay function was used to relate stimulus similarity to psychological distance (Nosofsky, 1986; Shepard, 1987):

(2) $$\eta_{ij}=e^{-d_{ij}}$$

where η*_ij_* represents the global similarity of a stimulus *i* to a stimulus *j*. The similarity value of a stimulus *i* to one exemplar of the category *X* is written η*_ix_*. The probability of responding with category *X* when faced with a forced choice between Categories *X* and *Y* was computed using the choice rule devised by Luce (1963):

(3) $$P\left(X∕i\right)=\frac{\underset{x\in X}{\sum }\eta_{ix}}{\underset{x\in X}{\sum }\eta_{ix}+\underset{y\in Y}{\sum }\eta_{iy}}$$

**Figure 2:**
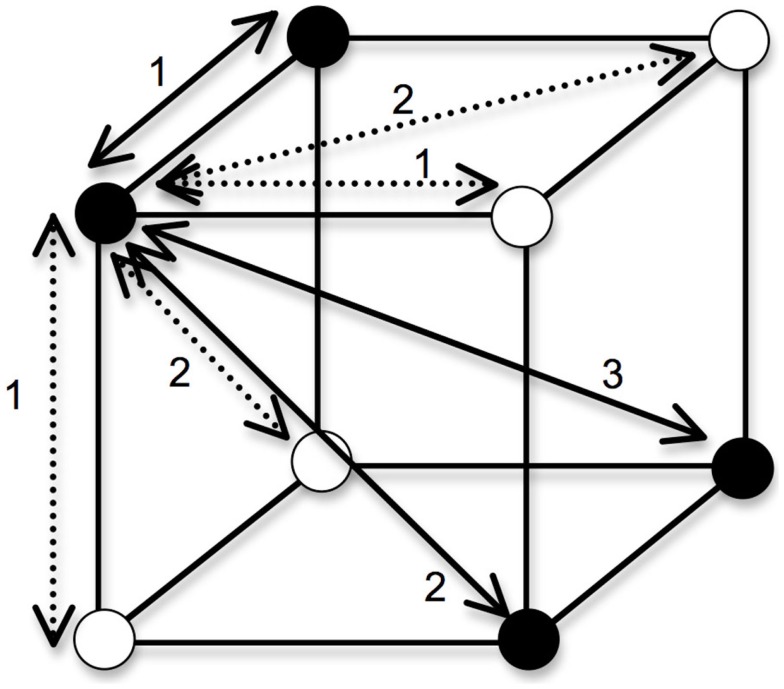
**Distances between objects in a Type II**. In generating the predictions of a simple exemplar model, we posit an XOR categorization in which the filled circles belong to one category, and the empty circles belong to a different category. Categorization accuracy is a function of the sum of the city-block distances of an object to the objects in its own category, divided by the sum of its distances to all objects. For the object in the front-upper-left of the cube, the within-category distances are shown by solid lines and the between-category distances shown by dashed lines. Not included in the figure is the distance of 0 that the object has to itself.

To obtain a measure of each concept’s complexity given the input distances, a single probability term was computed by taking the average of all *P*(*CorrectCategory/i*) over all stimuli *i*. Again, the predictions made by the full version of GCM (which includes a sensitivity parameter and attention weight parameters) give different quantitative predictions, but the ordinal predictions of our different study cases were identical to those made by RSM. The complexity of each categorization task resembles the notion of category intuitiveness defended by Pothos and Bailey (2009): a lower average probability corresponds to a less intuitive classification. For any of the objects that belong in the positive category, we can assume that every segment on which two objects differ adds a distance of 1 (see Figure 2), such that the predicted categorization accuracy for a sample close-similarity case is:

$$\begin{array}{llll} & p\left(X\right) & & \\ & =\frac{\exp\left(-0\right)+\exp\left(-1\right)+\exp\left(-2\right)+\exp\left(-3\right)}{\begin{array}{c} \left[\exp\left(-0\right)+\exp\left(-1\right)+\exp\left(-2\right)+\exp\left(-3\right)\right]\\ +\left[\exp\left(-1\right)+\exp\left(-2\right)+\exp\left(-1\right)+\exp\left(-2\right)\right] \end{array}}=0.61 & & \end{array}$$

Note that an object is compared to the four objects in the same category (including itself, hence the distance of 0), as well as the four objects in the other category. If we multiply the two RELEVANT dimensions’ distances by 2 (i.e., every edge has a distance of 2; this corresponds to our first study case in Figure 1), but keep the irrelevant dimension’s distances as they were in the first example, the categorization accuracy becomes:

$$\begin{array}{llll} & p\left(X\right) & & \\ & =\frac{\exp\left(-0\right)+\exp\left(-1\right)+\exp\left(-4\right)+\exp\left(-5\right)}{\begin{array}{c} \left[\exp\left(-0\right)+\exp\left(-1\right)+\exp\left(-4\right)+\exp\left(-5\right)\right]\\ +\left[\exp\left(-2\right)+\exp\left(-3\right)+\exp\left(-2\right)+\exp\left(-3\right)\right] \end{array}}=0.79 & & \end{array}$$

If we multiply the IRRELEVANT dimension’s distance by 2 (this corresponds to our second study case in Figure 1), but keep the relevant dimensions’ distances as they were in the first example, the categorization accuracy becomes:

$$\begin{array}{llll} & p\left(X\right) & & \\ & =\frac{\exp\left(-0\right)+\exp\left(-2\right)+\exp\left(-2\right)+\exp\left(-4\right)}{\begin{array}{c} \left[\exp\left(-0\right)+\exp\left(-2\right)+\exp\left(-2\right)+\exp\left(-4\right)\right]\\ +\left[\exp\left(-1\right)+\exp\left(-1\right)+\exp\left(-3\right)+\exp\left(-3\right)\right] \end{array}}=0.61 & & \end{array}$$

Finally, if we multiply ALL of the distances by 2 to represent a case in which we uniformly increase the dissimilarity between objects, we get:

$$\begin{array}{llll} & p\left(X\right) & & \\ & =\frac{\exp\left(-0\right)+\exp\left(-2\right)+\exp\left(-4\right)+\exp\left(-6\right)}{\begin{array}{c} \left[\exp\left(-0\right)+\exp\left(-2\right)+\exp\left(-4\right)+\exp\left(-6\right)\right]\\ +\left[\exp\left(-2\right)+\exp\left(-4\right)+\exp\left(-2\right)+\exp\left(-4\right)\right] \end{array}}=0.79 & & \end{array}$$

As such, for the Type II categorization, this simplified exemplar model predicts that categorization accuracy is affected by distances on the relevant dimensions, but not the distances on the irrelevant dimension. Again, this *pattern* of results does not change by considering a more complex exemplar model such as one that includes a sensitivity parameter, with similarity modulated by *e^−cd^* instead of *e^−d^*. For instance, applying *c* = 1.5 to the model, the respective values are 0.70 and 0.91 instead of 0.61 and 0.79; the respective values for *c* = 2 are 0.79 and 0.96. However, it must be noted that for more extreme values such as *c* = 5 the values are 0.99 and 1. In general, the model is less affected by the manipulation of distance as *c* increases and overall categorization accuracy reaches a ceiling level of performance. The reason is that altering *c* values does not modulate the 0 distance, hence the similarity of one object to itself becomes progressively dominant in the equation and the probability of responding with the right category increases.

The results of our simulations of RSM are indicated in the predictions summarized in Figure 1 (under the column “Find diff. btw,” i.e., finding differences between categories, a process that seems to describe in simple terms the behavior of RSM). Contrary to a learning process that focuses on finding similarities between sub-categories (under the column “Find sim. within”), which would predict more difficult categorization when increasing the distance between objects on the relevant dimensions, RSM predicts that learning is made easier by that same increase. Note that this is not necessarily an intuitive prediction even for those knowing well the principles governing the exemplar model.

In Figure 1, increased distances are represented by the blue arrows that simulate the stretching of the original cube on the left. A second prediction relates to increasing the distance between the features of the irrelevant dimension (this is represented by the second line in the table, where the single blue arrow stretches the cube to make it deeper). As shown by the above calculations, increasing the distance between the features of the irrelevant dimension has no effect on the RSM output. However, if a learning process predominantly uses similarities to form a category representation, this increase would clearly affect categorization by hindering the formation of the sub-categories. Our method is therefore based on manipulating the distances between categories and sub-categories to test these two sets of predictions.

## Materials and Methods

We seek to understand the effects of increasing the salience of the diagnostic dimensions in a single disjunctive artificial classification task. This manipulation has two simultaneous effects: increasing the distance between members of the same category and increasing the distance between members of opposite categories. Like the 5–4 category structure (Medin and Schaffer, 1978; Smith and Minda, 2000), we hypothesize that this manipulation can operate as a critical benchmark test for categorization models.

### Participants

The participants were 54 undergraduate students at the University of Franche-Comté who received course credit in exchange for their participation. The participants were randomly assigned to the experimental conditions. Informed consent was obtained from all participants, the experiments conformed the relevant regulatory standards, and the experiment was approved by the Université de Franche-Comté.

### Apparatus

The stimuli were geometric objects that could vary along three dimensions with each dimension presenting four different values, from which two values were picked for building a set of objects to be categorized: color (any two of the following: 20, 40, 60, and 80% gray, on the RGB 0-255 range), shape (extra simple, simple, complex, and extra complex fractals; the fractals and their shape are detailed later), and size (11 cm × 8.5 cm, 8 cm × 6.4 cm, 6 cm × 4.7 cm, 4.5 cm × 3.5 cm, which is a respective diminution of 33% from one size to the next one).

**Figure 3:**
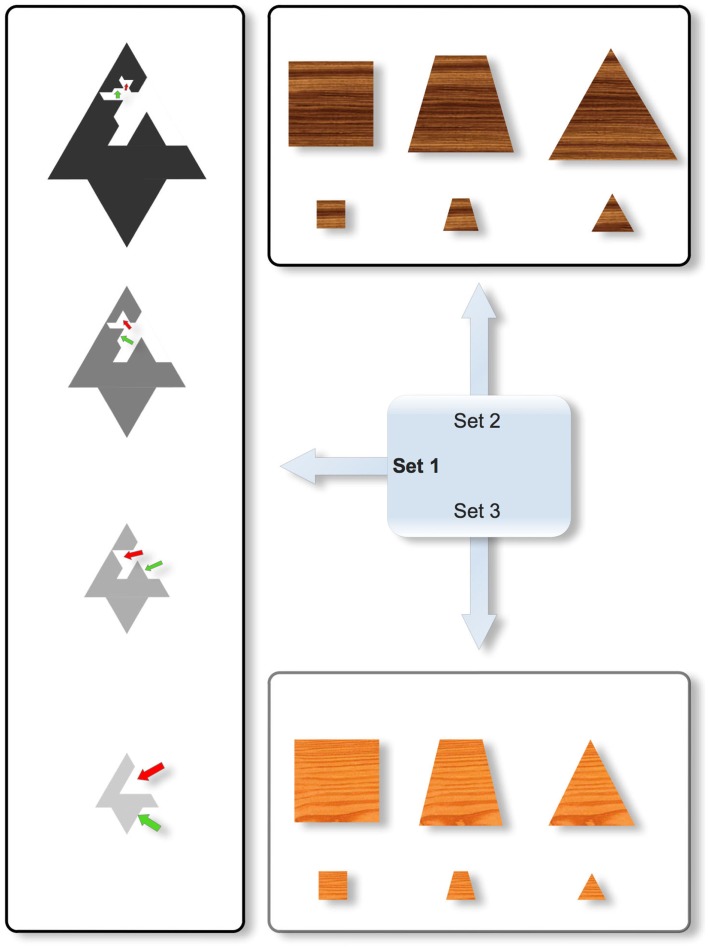
**Three different sets of stimuli differing on the possible interaction between their constituent size and shape dimensions**. Only Set 1 was used in the experiment. In Set 1 (Left), the four stimuli differ in color, size, and shape. Note that for a given size, the different shapes necessarily possess constant areas and constant convex envelopes (note that this cannot be directly observed in the figure because no stimuli in Set 1 have the same size, but for different shapes, it is easy to figure out that the rubber band would wrap around the shapes with the same length). The red and green arrows that show the displacement of some parts were not part of the stimuli that were shown to participants. In Set 2 (Top), the stimuli vary in shape from left to right by keeping the area constant, but in that case, the convex envelope surrounding the object increases from left to right so that the perceived size might vary according to changes in shapes. As a result, the top left square might seem more similar to the bottom right triangle than the top right triangle in comparison to the bottom left square (whereas the ratios of the areas for the two respective pairs are equal). In Set 3 (Bottom), the shapes vary from left to right while keeping the height and the base constant. As a result, the top left square might seem further from the bottom right triangle than the top right triangle in comparison to the bottom left square (because the ratios of the areas for the two respective pairs are unequal).

In order to limit the dependence between shapes and sizes (two dimensions that have been shown to interact and govern performance, see Love and Markman, 2003; Mathy and Bradmetz, 2011), a new kind of stimuli was devised for this study. The intention was to create shapes with equivalent areas and equivalent convex envelopes for a given size. We thought that psychologically speaking, areas and convex envelopes can be more important than perimeter to judge the size of an object. Effectively, the tentative Set 2 in Figure 3 did not seem appropriate for our study, because when the stimuli vary in shape from left to right, and the area remains constant, the convex envelope increases from left to right. As a result, the perceived size might vary according to changes in shape: for instance, the top left square might seem closer to the bottom right triangle than the bottom left square in comparison to the top right triangle (whereas the ratios of the areas for the two respective pairs are equal). Such an effect might facilitate the formation of a Type II concept based on the top left and bottom right objects. In Set 3, the shapes vary from left to right by keeping the height and the base constant. As a result, the top left square might seem further apart from the bottom right triangle than the bottom left square is from the top right triangle (because the ratios of the areas for the two respective are unequal). This effect can also be particularly detrimental to studying similarity effects in the formation of a disjunctive concept. Accordingly, both Sets 2 and 3 were rejected in favor of Set 1. The four stimuli in Set 1 differ in color, size, and shape independently (the four respective values are shown in the four objects of Figure 3, a subset of the 4 × 4 × 4 = 64 possible objects; from bottom to top, the four objects become darker, larger, and more complex). Starting from the simplest object (bottom), the more complicated objects were built recursively by cropping a triangle in the middle of the shape and by displacing the cropped triangle on an opposite segment (the four displacements are indicated by the red and green arrows in Figure 3; note that these arrows were not seen by the participants). For a given size, this resulted in the different shapes having constant areas and constant convex envelopes. Effectively, by imagining a rubber band analogy wrapping around the objects to compute the convex envelope, the length, and shape of the elastic band is identical from one shape (i.e., complexity) to another *for a given size*. The four different values for each dimension were respectively coded from 1 to 4 to express the decrease in size, the increase of complexity in shape, and the decrease in darkness. Although the stimuli do not equate for perimeter as shapes vary, the potential for psychological interactions between area and brightness, and between area and size, were deemed to be more problematic than between perimeter and either size or brightness.

### Design

The Type II categorization requires two diagnostic/relevant dimensions, one irrelevant dimension, two values per dimension, and eight stimuli (four positive, four negative). A logical rule such as “*ab* or *a*′*b*′” is the signature for a Type II structure, with the third dimension being irrelevant. For that reason, a first factor determined whether the concept was Shape-irrelevant, Size-irrelevant, or Color-irrelevant. Once the irrelevant dimension was chosen (e.g., a Shape-irrelevant condition for the Irrelevant factor), we balanced the two remaining choices for associating the chosen set of objects to the positive and negative categories: four of the eight objects were either assigned to the positive category [e.g., for one participant, the concept was “(*large* and *dark*) or (*small* and *light*)”] or the four other objects were assigned to the positive category [e.g., for another participant: “(*small* and *dark*) or (*large* and *light*)”]. This manipulation was the Balance of categories factor. The four dimension values were recoded by their rank (1, 2, 3, and 4).

The second main manipulation concerned the opportunities for stimulus discrimination: for each dimension, the differences in size, color, and shape values were one of the three following: (a) 2 - 1 = 1 (i.e., a difference of 1 resulting from using the second and first value of one dimension), (b) 3 - 1 = 2, or (c) 4 - 1 = 3. This manipulation was called the Distance between dimensions factor. This choice means that not all pairs of values were studied, to lower the number of experimental conditions (for instance, there were no 3 - 2 = 1 or 4 - 3 = 1 conditions, etc.; note that the first value for each dimension was always picked as a base, so only the second value freely varied). Another constraint was that the two relevant dimensions always had the same distance between their features. Overall, the between-subject design involved:

Overall, the between-subject design involved 3 (Irrelevant factor) × 2 (Balance of categories) × 3 (Distance between relevant dimensions) × 3 (Distance between irrelevant dimensions) = 54 different conditions.

### Procedure

The 54 participants were assigned to one of the 54 different conditions just described for the category learning task. They all completed a pre-categorization similarity rating task, a category learning (classification) task, and a post-categorization rating task^2^. During the pre- and post-categorization similarity rating tasks, the participants were required to judge the similarity between each pair of the eight objects they were assigned for the classification task. This part was completed by rating the similarity of pairs of objects on a 1–9 scale with 1 indicating “not very similar at all” and 9 indicating “highly similar.” Assuming that the participants would give symmetric similarity ratings, they were given (8 × 7)/2 = 28 judgments for pairs of objects that were presented side-by-side simultaneously until a response was made. During the post-categorization phase, the procedure was identical to the pre-categorization task except that the participants were instructed to try to provide similarity judgments that were independent from both the pre-categorization phase and the classification task. That is, they were asked to judge the similarity between the objects without considering the preceding experience they had with the stimuli, so that their judgments could freely vary from their previous ones. Each of the similarity rating tasks required approximately 5 min.

For the category learning task, each participant was assigned a single Type II concept that could be described by one of the 54 conditions described above. The concept was learned by trial and error in a single session that lasted about 15 min. Testing was preceded by a brief explanation about how to sort the stimuli (by pressing 1 or 0 for the respective positive and negative categories). Feedback was displayed at the bottom of the screen (for 2 s) to indicate whether a response was correct or incorrect. To keep track of the participant’s “score,” one point was added to a progress bar for each correct response. The number of increments in the progress bar was equal to four times the length of the training sample, that is, 4 × 8 = 32. The criterion of 4 × 8 was identical to the one used in the pioneering study of Shepard et al. (1961). Participants had to correctly categorize the stimuli on four blocks of eight stimuli – that is, they had to fill up the progress bar to terminate the experiment. Participants were told that the classification task would end once the entire progress bar filled up. A learning criterion (unknown to the participant) was set to 2 × 8 = 16 consecutive correct responses. The response times (RTs) were measured during the last 16 correct responses to determine whether differences in learning times persisted with the learned concept compared to the practice items. When an incorrect response was made, all points scored thus far were lost (although the progress bar only went back to 16 points for cases in which the participant succeeded in getting more than 16 correct responses in a row). This means that participants had to correctly categorize the stimuli on two consecutive blocks to reach the learning criterion, and then again on two other consecutive blocks to terminate the experiment. Using consecutive blocks insured that the participants were learning all the stimuli rather than a subset of them. We noticed in several pretests that resetting the progress bar to zero when more than half of the progress bar was completed was frustrating for participants. Resetting the bar to the halfway point when they made an error after 16 correct responses encouraged the participants to continue with the experiment. The learning criterion was established so that RTs would be interpretable, but it did not mean that the participants had perfectly memorized the category members or that they could perfectly classify the rest of the stimuli. A few errors could still occur after the so-called learning criterion.

Stimulus objects were presented one at a time in the upper half of the computer screen, and the lower part of the screen was reserved for the feedback. The stimuli were randomly permuted within all blocks, with the constraint that the first stimulus in each block was different from the last one in the previous block. The categorization of one stimulus object was considered a trial.

The dependent variables were the number of blocks required to reach the learning criterion, the proportion of errors measured before the progress bar reached eight correct responses in a row (which reflected performance before the concept was acquired), and the RTs measured when the progress bar was above 16 (the rationale being that the analysis of RTs is most readily interpretable when a correct response is given, otherwise, without more refined models, short RTs can either represent fast processing or guessing responses). In order to avoid inappropriate inferential statistics given the positively skewed distribution of the RTs, RTs were transformed by the natural logarithm (LN).

Our hypothesis was that harder conditions should provoke an increase in the number of blocks required to reach the learning criterion, an increase in the number of incorrect responses, and longer RTs (reflecting more difficult processing of the stimuli because of a more complicated retrieval of exemplars, or because of the use of a less effective rule that a participant would find more difficult to encode during the learning process).

## Results

### Saliency effects

We first investigated whether differences in performance were due to dimension salience. Regarding the number of blocks required by the participants to reach the learning criterion, we observed no differences between the three main Size-, Shape-, and Color-irrelevant conditions, with an average of about 26 blocks (SD = 15). The irrelevant dimension also did not result in a significant difference in the proportion of errors per block, with an average of about 0.40 errors per block (SD = 0.19) across these three conditions. A difference, however, was observed in the last two blocks for RTs, with an average of about 1.5 s (SD = 0.87) to classify the objects in the Color-irrelevant condition compared to 1.7 s (SD = 1.01) for the two Color-relevant conditions. To gain sufficient statistical power and to describe the whole distribution, a preliminary analysis included the RTs from 1008 correct trials across the 54 participants whenever they had accumulated 16 previous correct responses (most of the time, this corresponded to the last two blocks, unless the participant made a mistake that reset the progress bar). A one-way ANOVA showed that there were significant differences across the three Size-, Shape-, and Color-irrelevant conditions on RT, *F*(2,1005) = 4.5, *p* = 0.011, η^2^ = 0.009, although the effect size was low. The same analysis on LN(RT) was slightly more powerful, *F*(2,1005) = 5, *p* = 0.007, η^2^ = 0.01. The *post hoc* comparisons (Newman–Keuls) confirmed the significant difference of the Color-irrelevant condition compared to the two other conditions. Overall, the choice of the relevant dimensions seemed to have little effect on performance when the whole RT data (after learning criterion) was taken into consideration. We then run a more accurate mixed model analysis (appropriate in the presence of the correlated errors that can arise from a data hierarchy), using subject number as a random factor, stimulus type as a repeated variable, and irrelevant condition as the main factor, using a maximum likelihood estimation method, and again, using LN(RT) as the dependent variable in the last two blocks. The RT were previously averaged in order to generate 432 data points from the 54 subjects × 8 stimulus types table. The subjects were embedded in the three Size-, Shape-, and Color-irrelevant conditions. The analysis showed a significant fixed effect of the irrelevant condition, *F*(2,422), 5.34, *p* = 0.005, with pairwise comparisons showing that the Color-irrelevant condition was significantly lower than the two Color-relevant conditions.

From the exemplar model perspective, the slightly faster processing of Color-irrelevant concepts suggests that Size and Shape were the most salient dimensions for subjects, especially when combined together as relevant conditions. Remember that the exemplar model falls in the “Find diff. btw” column in Figure 1, so when the relevant dimensions are spread out, categorization is predicted to be easier. In this perspective, the faster processing that we observed may be due to increased dimension salience increasing the perceived distance between exemplars, which facilitates an exemplar-based classification strategy. This exemplar-based explanation tends to be confirmed by the analysis conducted on the pre-categorization similarity rating task using multi-dimensional scaling (MDS): Shape was found to be the most salient dimension for 50 participants, followed by Size as the second most salient dimension for 32 participants.

In addition to the MDS-based analysis conducted on the pre-categorization similarity rating task, a simpler non-MDS measure of dimension salience was assessed by computing the difference in similarity ratings when the objects shared versus did not share one feature, which was conducted for each dimension across all trials, that is 54 subjects × 28 pairs. The pairs were then averaged for each dimension and when the objects shared versus did not share one feature, which generated 324 conditions from the 54 subjects × 3 dimensions × 2 shared-feature combination. We found a similar ranking of the dimensions, with the greatest difference between the mean dissimilarities (1.9 versus 5.2) for the Shape dimension, 3.9 versus 3.7 for the Size dimension, and 3.4 versus 4.1 for the Color dimension. The two-way repeated measures ANOVA on the dissimilarity ratings showed a significant effect of the shared- versus not shared-feature factor, *F*(1,53), 371, *p* < 0.001, $\eta_{p}^{2}=0.88$, a significant effect of the Dimension factor, *F*(1.7,88.1), 130, *p* < 0.001, $\eta_{p}^{2}=0.71$ (the degrees of freedom were corrected using the Greenhouse–Geisser estimates, because the Mauchly’s test indicated that the assumption of sphericity had been violated), and a significant interaction between the two factors, *F*(1.7,88.1), 130, *p* < 0.001, $\eta_{p}^{2}=0.71$.

### Distance effects

**Figure 4:**
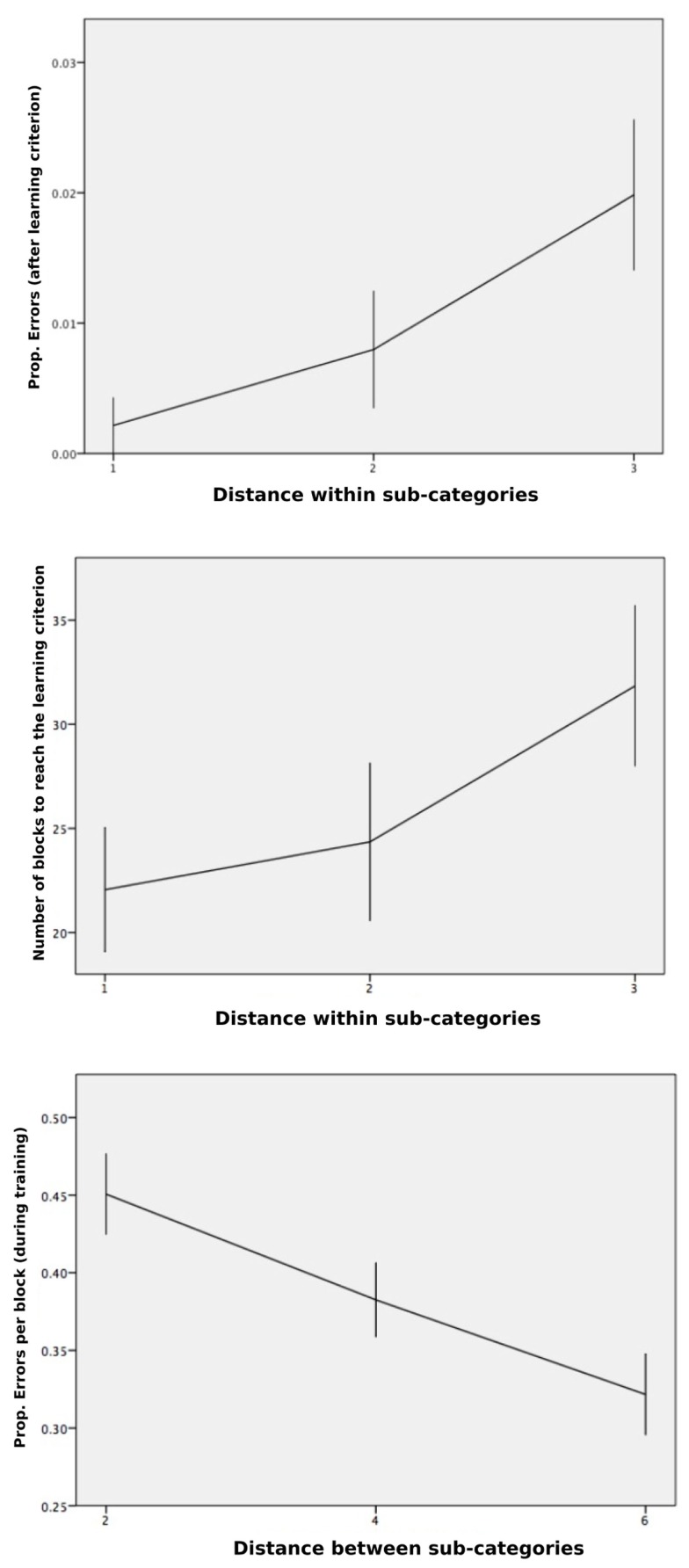
**Classification performance**. Top, shows an increase in the mean proportion of errors per block, computed for the blocks that passed the learning criterion, given the distance on the irrelevant dimension (i.e., greater dissimilarity within clusters). Middle, shows an increase in the mean number of blocks to learning criterion, given the distance on the irrelevant dimension. Bottom, shows a decrease in mean RTs (transformed by a log function) after the learning criterion was reached, as the distance increased on the relevant dimensions (i.e., greater dissimilarity between cluster). Error bars are ±1 SEM.

We then computed the distance between the values of the relevant dimensions (Between-Clusters Distance) and the distance between the values of the irrelevant dimension (Within-Cluster Distance). Following Pothos et al. (2011), who targeted a basic arithmetic description of the similarity structure in their geometric approach, distance measures were averaged across all stimulus pairs belonging to a given type of relationship (between clusters, within clusters). Our objective was to focus on the independent contribution of both types of distances on each of the dependent variables. The multiple regression on the proportion of errors (computed by block across participants) against both distances did not lead to any significant fit [*F*(2,1235) = 0.4, NS] when the trials taken into account were restricted to those that preceded the learning criterion. However, when the same analysis was run for the trials that passed the learning criterion (remember that committing an error was still possible after the so-called learning criterion), the multiple regression on the proportion of errors against both distances indicated a significant relation [*F*(2,165) = 4.2, *p* = 0.016, *R*^2^ = 0.05], and led to a significant standardized coefficient for the Within-Cluster Distance (β = 0.218, *p* = 0.005). The mean proportions of errors for Within-Cluster Distances equal to 1, 2, or 3 were respectively 0.002, 0.008, and 0.020, indicating that the greatest distance caused more errors after the learning criterion. Figure 4 (top) shows this significant increase of classification errors with increasing distance within sub-categories, after participants reached the learning criterion.

Another multiple regression on the total number of blocks to reach the learning criterion against both distances led to a nearly significant standardized coefficient for the Within-Cluster Distance (β = 0.269, *p* = 0.051), ending up with a global non-significant fit [*F*(2,51) = 2.28, NS]. Figure 4 (middle) shows the slowing down of concept induction with increasing distance within sub-categories, although the effect was short of significance (*p* = 0.051), likely because of the high standard errors within each condition and fewer degrees of freedom. In order to increase statistical power for our analysis on the number of blocks, we restricted the analysis to the two extreme conditions (Within-Cluster Distance = 1 and Within-Cluster Distance = 3), which correspond to about 22 and 32 blocks, respectively (we note that a difference of 10 blocks indicates that a substantial number, 80, more stimuli were needed to reach the learning criterion when the distance within sub-categories was set to 3 relative to 1). By removing the Within-Cluster Distance = 2 condition, the analysis still fell short of significance, *t*(35) = 1.99, *p* = 0.054.

To summarize, the positive signs of the coefficients (0.218 and 0.269) indicated respectively more errors and more difficult learning for an increased distance within sub-categories; this confirms that categorization is hindered when similarities are difficult to find. The simplified exemplar model that we presented does not predict these influences of the salience of the irrelevant dimension on categorization.

A last regression carried out on LN(RT) was significant [*F*(2,1005) = 7.84, *p* < 0.001, *R*^2^ = 0.02]. Although the effect size was again small, this result allowed us to address the similarity-dissimilarity competition issue. This analysis revealed a significant coefficient for the Between-Cluster Distance (β = −0.112, *p* = 0.001), with the negative sign of the coefficient indicating lower RT as distance on the relevant dimensions increased. This pattern is predicted by the exemplar model and indicates that increasing the distance between clusters favors performance and, therefore, a dissimilarity-based categorization process seems to override a similarity-based process. The respective means (see Figure 4, bottom) for the Between-Cluster Distances were equal to 2 (*M* = 0.45, SD = 0.49), equal to 4 (*M* = 0.38, SD = 0.46), or equal to 6 (*M* = 0.32, SD = 0.50).

### Pre- and post-categorization similarity rating tasks

We already mentioned that our MDS analysis showed that 50 participants judged Shape as the most salient dimension in the pre-categorization similarity rating task. For this pre-categorization rating task, the mean proportion of the scaled data (RSQ) across participants was 0.637 (SD = 0.177), with a minimum of 0.059 and a maximum of 0.916. Shape was identified as the first dimension in the MDS analysis, indicating that it was a more salient feature and suggests that the distance between two values on that dimension was perceived as being greater than the distances on the other dimensions. In the post-categorization task, the same trend was observed for 48 participants (see Table 1). Here, the mean RSQ across participants was 0.650 (SD = 0.148), with a minimum of 0.054 and a maximum of 0.890. One participant switched to Size, one switched to Color, and the judgment of two others produced a dimension that did not match any of the three Size, Shape, or Color dimensions. Conversely, two participants switched to Shape in the post-categorization task (cf. first crosstab in Table 1, where those numbers are found in the Shape row and the Shape column).

**Table 1:** **Crosstab showing how many times each of the dimensions (Size, Shape, or Color) could be mapped to the MDS results, during the pre- and post-categorization phases**.

Pre-cat task		Post-cat (first dim)				
		?	Size	Shape	Color	Total
	?	0	1	1	0	2
	Size	0	1	1	0	2
First dim	Shape	2	1	46	1	50
	Color	0	0	0	0	0
	Total	2	3	48	1	54
		**Post-cat (second dim)**				
		**?**	**Size**	**Shape**	**Color**	**Total**
	
	?	4	5	1	2	12
	Size	3	21	2	6	32
Second dim	Shape	0	1	0	1	2
	Color	4	0	0	4	8
	Total	11	27	3	13	54

*Metric MDS with interval data was run using SPSS, with three dimensions requested. The ? symbol means that the dimension did not matched any of the Size, Shape, or Color dimensions*.

Regarding the second most salient dimension, in the pre-categorization similarity rating task, 12 participants produced patterns of similarities that could not be mapped to any of the Size, Shape, or Color dimensions, 32 patterns corresponded to Size, 2 to Shape, and 8 to Color. During the post-categorization task, regarding the second most salient dimension, 11 patterns could not be determined, 27 corresponded to Size, 3 to Shape, and 13 to Color (cf. the crosstab in Table 1).

The categorization phase did not strongly influence the perceived distances during the post-categorization phase, especially for Shape. Moreover, among those for which there was a change in the ranking of the dimensions from the pre- to the post-categorization task, only nine participants judged the dimensions that were relevant during the categorization task as the most salient ones in the post-phase on occasions when they were not their spontaneous choice during the pre-categorization task (note that this information cannot be taken out from the crosstabs). This suggests that the classification task had little effect on what dimension was judged as salient.

**Figure 5:**
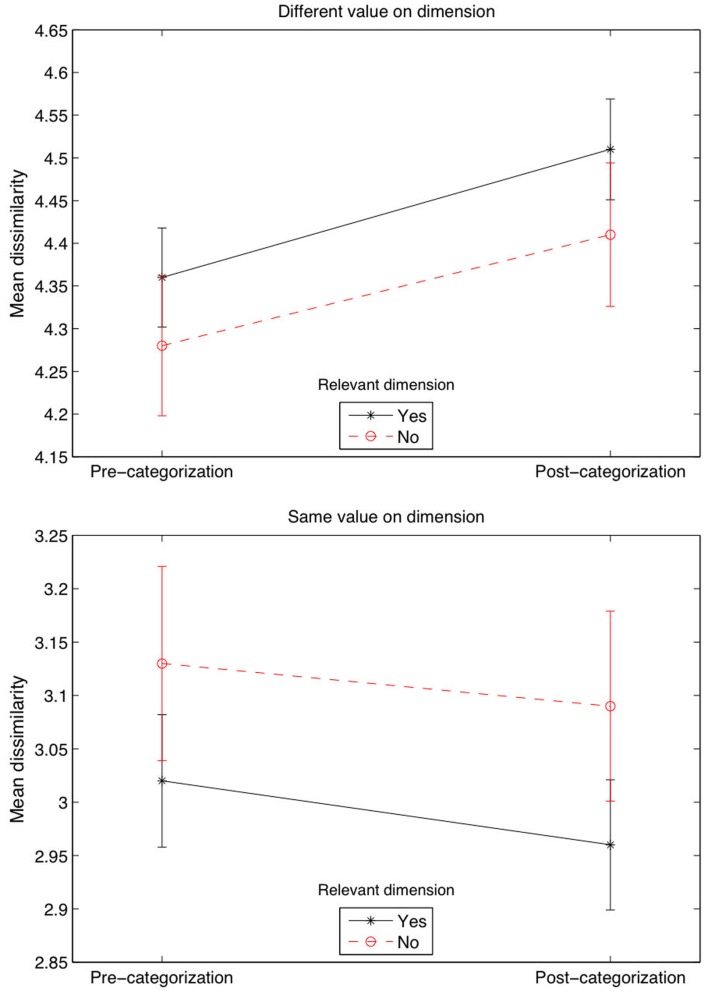
**Mean dissimilarity ratings across all trials**. Error bars are ±1 SEM.

Again, we included a simpler non-MDS measure of dimension salience based on the difference in similarity ratings when the objects shared versus did not share a feature, conducted for each dimension across all trials. Our objective was to see whether this difference changed from the pre-categorization task to the post-categorization task, and in particular, if this difference could be modulated by a Relevant variable (for which we coded Relevant = “yes” if the dimension was one of the two relevant dimensions, and Relevant = “no” otherwise), first across all trials. The ANOVA across all trials that included Phase (pre- versus-post), Relevant, and same Dimension Value (yes versus no, meaning that a feature was shared versus not shared) as three fixed factors. The analysis showed a significant effect of same Dimension Value [*F*(1,9064) = 637, *p* < 0.001] and a Relevant × same Dimension Value interaction [*F*(1,9064) = 4.1, *p* < 0.05]. We also averaged the data and run a more appropriate three-way repeated ANOVA, which showed a significant effect of same Dimension Value [*F*(1,53) = 308, *p* < 0.001, $\eta_{p}^{2}=0.85$] and, in this condition, a Phase × same Dimension Value interaction [*F*(1,53) = 7.5, *p* = 0.009, $\eta_{p}^{2}=0.12$]. Figure 5 shows, for example, that the dissimilarity rating when two stimuli did not have the same value on one dimension was the highest when the dimension was relevant during the categorization phase (first plot, top right value), whereas the dissimilarity rating was lower when two stimuli had the same value on a relevant, compared to irrelevant, dimension (second plot, bottom right value). This indicates that the greatest divergence in similarity ratings occurred for the relevant dimensions, in line with previous research (Kurtz, 1996; Livingston et al., 1998; Goldstone et al., 2001). Dissimilarity was increased the most when objects differed on dimensions that were categorization-relevant rather than categorization-irrelevant. Likewise, similarity was increased the most when objects shared category-relevant compared to category-irrelevant dimension values.

## Discussion

Our results suggest that category learning is made slightly easier when both categories and sub-categories are perceptually teased apart. This finding presents a challenge for any model that does not allow for the possibility that the dissimilarity side (members of separate categories are more dissimilar when teased apart) sometimes predominates over the similarity side of a categorization process (members within the same category are less similar when teased apart). Independently, category learning was also found slightly more difficult with increased distances within sub-categories.

Our experiment also included two similarity rating tasks that led to similarity judgments quite disconnected from categorization performance. The reason for this disconnect might be that the similarity rating tasks are not the best method to assess how stimuli affect concept formation or how stimuli are affected by concept formation. For example, our pre-categorization similarity rating task showed that judgments about dimensional saliency only slightly affected the difficulty of concept discovery. Under the hypothesis that participants use hypothesis confirmation strategies (Hovland and Weiss, 1953; Bruner et al., 1956), Color-irrelevant concepts were perhaps found easier to use by our participants because Color was judged to be the least salient dimension. However, Shape (the most salient dimension) did not modulate the speed with which participants discovered the Shape-relevant concepts, whereas its saliency would have been expected to facilitate the discrimination of the categories. Nevertheless, the post-categorization similarity judgments showed greater perceived dimension salience that could have been favored by the discrimination process of the opposite categories. For example, by computing the mean dissimilarity ratings separated by relevant and irrelevant dimensions, our last analysis (Figure 5) showed that dissimilarity ratings considerably increased in the post-categorization task when the stimuli did not share the same dimension values on the relevant dimensions, a result that was suggested by previous research (Kurtz, 1996; Livingston et al., 1998; Goldstone et al., 2001).

We now further discuss the category learning task. Many categorization models do not share the same assumptions. Traditionally, they are contrasted as active/explicit hypothesis testing versus passive/implicit stimulus-association, akin to a rule-based versus exemplar-based category learning process contrast (Estes, 1994; Ashby et al., 1998; Medin et al., 1993; Hahn and Chater, 1998; Smith et al., 1998; Ashby and Ell, 2001, p. 5; Hampton, 2001). As a result, opposite predictions can be made for learning a disjunctive concept such as Shepard et al.’s (1961) Type II (which was administered to participants of the present study), especially when the sub-categories are teased apart. We made several predictions based on which category learning process is favored by individuals (i.e., finding differences between categories or finding similarities between sub-categories). Our experiment stretched the two relevant dimensions in a Type II concept, a manipulation that resulted in more dissimilarity both within and between categories, thus introducing a similarity-dissimilarity competition. A second manipulation increased dissimilarities within sub-categories in the Type II concept by stretching the irrelevant dimension.

To clarify, we consider that seeking similarities between members of a category is more typical of a rule-based process. This process recalls a strategy based on positive information in the classical view of categorization (Bruner et al., 1956). Effectively, it has long been thought that the formulation of a rule is mostly based on describing the category members – not necessarily by opposition to other categories (one can define a cat without necessarily opposing cats to dogs or even less to crocodiles). Increasing similarity might affect the emergence of rules because with greater density, shorter rules are possible, which reduces the space of hypotheses (conversely, a greater number of possible rules arises as the number of features increases). For instance, the concept of cyclops is quite easy to acquire because the decision boundaries are confined to one-eyed creatures. This is not incompatible with the idea that categorization rules develop as a result of corrective feedback, given that both positive and negative feedback can be provided for someone attempting to define what a cyclops is (for instance, a *yes* response for the judicious question “Does a cyclops have one eye?”). Similarity also simplifies relational concepts: it seems reasonable to find some similarities between the sub-categories “white cats” and “black dogs” as you can easily imagine someone being sensitive to this limited association of pets and colors. However, it seems less intuitive to guess that someone has an exclusive preference for “white pieces in chess” and “black keys on a piano,” because chess and piano parts are completely unrelated domains. On the contrary, an exemplar model simulating the learning of the Type II model predicts poorer performance when there are more similarities among between-category clusters, primarily because of the corollary decrease in dissimilarities between opposing categories. This opposition between the models is consistent with previous observations that a rule-based model (e.g., General Recognition Theory) better describes categorization with closer stimuli, whereas an exemplar model (e.g., General Context Model) better describes categorization with distinct stimuli (Rouder and Ratcliff, 2004, p. 78).

Our main result is that increased distances on the relevant dimensions (1) facilitate the use of a Type II concept (Figure 4, bottom panel), suggesting that participants relied more on using dissimilarities between opposite categories than using similarities between sub-categories. This result corroborates the prediction made by the exemplar model. However, we also found that increasing the distance within sub-categories tends to: (2) slow concept discovery (Figure 4, middle panel) – although the effect fell short of significance; and (3) lead to more classification errors after participants reached our learning criterion (Figure 4, top panel). Although we supposed that the concept was solidly acquired after two consecutive blocks, this was not the case when the distance within sub-categories was the highest. This last result rather points to a rule-based account for which diminished similarities within sub-categories hinders learning. A basic exemplar model is not able to fit this particular result.

A possible conclusion is that the contrasting of categories is not the only favored process by which separable categories are acquired, but that comparing and abstracting the features within-category also matters. Such a conclusion follows other research on the influence of item juxtapositions on learning (Hammer et al., 2009; Andrews et al., 2011). This would support the idea that there is a tendency for humans and animals to readily group together things that look alike (Urcuioli, 2001). This conclusion also supports the observation that presenting successive exemplars by category (i.e., blocking) can sometimes result in better learning, in opposition to interleaving exemplars of different categories, although it is more correct to say that category structure modulate interleaving and blocking advantages (Gagné, 1950; Clapper and Bower, 1994; Goldstone, 1996; Kornell and Bjork, 2008; Mathy and Feldman, 2009; Carvalho and Goldstone, 2012). However, although there were different theoretical justifications for predicting that increasing distances between clusters in a Type II categorization would either make the categorization harder or easier, our experimental design still has limitations. In particular, our results are likely not entirely independent of the set of stimuli that were used. There might well be situations when similarity and dissimilarity are not simply linearly related to the physical dimensions, and it might be difficult to predict which of two opposing effects wins out. Future studies could directly target the same experimental design with different stimuli using finer parametric control over dimensional similarity. It is possible that future such experiments would reveal non-monotonic influences of dimensional similarity on categorization accuracy.

One promising model that would account for influences of both within- and between-cluster similarity on categorization accuracy is SUSTAIN (Love et al., 2004). The results do not necessarily require a hybrid model that includes distinctly separable processes for rule-based and exemplar categorization (e.g., Nosofsky et al., 1994b; Smith and Sloman, 1994; Erickson and Kruschke, 1998; Anderson and Betz, 2001; Rosseel, 2002). SUSTAIN occupies a position between exemplar models that encode every observed example from all presented categories and prototype models that summarize a category by a single central tendency. SUSTAIN assumes that categories are represented by a set of clusters which capture regularities both within and between categories. For example, when learning a rule-plus-exception category, SUSTAIN creates one cluster to capture the rule-following items and a separate cluster to capture the exception. New clusters are recruited based on the ability of existing clusters to accommodate presented items. In the case of a XOR categorization, two clusters would be created per category. Within each of the clusters, increasing the psychological distance between items hurts categorization efficiency because the recruited cluster centroid will fit the items within the cluster less closely. At the same time, increasing the psychological distance between clusters helps categorization efficiency because SUSTAIN will more easily establish separate cluster encodings for the separate clusters of the XOR problem. For models like SUSTAIN that create separate representations for the different sub-clusters that comprise the XOR category (see also the Rational Model of categorization of Anderson, 1991), the easiest categories to learn will be those that have the highest similarity within the clusters and the greatest separation between clusters – exactly the trends identified by our results.

Generalizing beyond specific models of categorization, our results emphasize the importance of going beyond a simple characterization of categorization difficulty in terms of within- and between-category similarity. Researchers have previously described categorization as being made increasingly difficult as a function of between-category similarity and increasingly easy as a function of within-category similarity (e.g., Rosch and Mervis, 1975; Goldstone, 1996). Our results suggest that this generalization should be made more nuanced. Within-category similarities can both hinder and help categorization. In particular, within-category similarities that allow tighter sub-categories to form can benefit categorization, but within-category similarities also can hinder categorization if they lead to confusing separate clusters that must remain identifiably different if the category is to be successfully acquired.

## Conflict of Interest Statement

The authors declare that the research was conducted in the absence of any commercial or financial relationships that could be construed as a potential conflict of interest.

## Footnotes

^1^See Goodwin and Johnson-Laird (2011) for a shorter description using the *or else* connector.

^2^Post-categorization testing was carried out to test the idea that categorization can influence perception (Whorf, 1956; Goldstone, 1994a; Gureckis and Goldstone, 2008).
